# Semantic content of visual stimuli has a greater impact on associative equivalence learning than stimulus modality

**DOI:** 10.3389/fnhum.2026.1882538

**Published:** 2026-07-22

**Authors:** Kálmán Tót, Noémi Harcsa-Pintér, Gabriella Eördegh, Balázs Bodosi, Attila Nagy

**Affiliations:** 1 Department of Physiology, Albert Szent-Györgyi Medical School, University of Szeged, Szeged, Hungary; 2 Department of Theoretical Health Sciences and Health Management, Faculty of Health Sciences and Social Studies, University of Szeged, Szeged, Hungary

**Keywords:** cognitive functions, human, visual, audiovisual, stimulus semantic content, equivalence learning

## Abstract

**Introduction:**

Associative equivalence learning, the ability to form connections between different stimuli based on shared outcomes, plays a fundamental role in human cognition. In this study, we investigated how stimulus modality (visual vs. audiovisual) and the semantic content of the visual stimuli affect performance in associative equivalence learning.

**Methods:**

We applied four tests (FaceFish, Polygon, SoundFace, and SoundPolygon) based on the Rutgers Acquired Equivalence Test, in a sample of 117 healthy adult participants. The tests differed in modality and semantic content of visual stimuli. All tests were divided into an acquisition phase, where participants learned associations based on shared outcomes, and a test phase, where they retrieved the learned associations and applied the acquired equivalence to new stimulus pairs.

**Results:**

Our results revealed that visual semantic content had a stronger and more consistent effect on performance than stimulus modality, particularly in the acquisition phase. Audiovisual presentation offered some facilitative effects (lower error ratios), especially when combined with visual stimuli with higher semantic content, but did not compensate for the limitations of simplified stimuli.

**Discussion:**

These findings support the critical role of visual stimulus features in shaping learning efficiency and suggest that the benefits of multisensory input may depend on the semantic content of visual stimuli. The results also validate previous findings obtained from smaller samples and highlight directions for future research, including developmental, multisensory, and neurophysiological investigations of associative learning.

## 1 Introduction

Equivalence learning is an essential cognitive function in humans, where two or more discrete and often different percepts (antecedents) are linked together based on a shared outcome or consequence (Ward-Robinson and Hall, 1999; Molet et al., 2012). This specific kind of associative learning can be investigated with the Rutgers Acquired Equivalence Test (RAET or FaceFish test) (Myers et al., 2003; Shohamy and Wagner, 2008), in which cartoon faces (antecedents) are associated with fish (consequents). This is a visual test that can be divided into two main phases. The feedback-controlled acquisition phase depends mainly on the function of the basal ganglia-frontal cortex circuits (Myers et al., 2003; Moustafa et al., 2009; Moustafa et al., 2010). In the acquisition phase, participants match two visual stimuli (an antecedent with one of two consequents) and receive feedback about the correctness of their choices. Through this trial-and-error learning, an equivalence is formed between the antecedents that share the same consequent. The test phase, where no more feedback is provided about the correctness of the choices, depends primarily on the function of the hippocampus (Cohen et al., 1999; Gogtay et al., 2006). In the test phase, participants recall the previously learned associations (retrieval) and create new ones based on the acquired equivalence (generalization or transfer). Measures of acquired equivalence learning, such as the Rutgers Acquired Equivalence Test and its variants, are useful tools for assessing associative learning, flexible retrieval, and generalization in both typical and atypical cognitive function. Because different task phases may rely partly on frontostriatal/basal ganglia and hippocampal mechanisms, examining the influence of stimulus modality and semantic content may help clarify the role of these brain structures in different clinical populations.

In its original form, RAET applies stimuli with high semantic content and different colors, which can help participants learn the associations by evoking emotions, cognitive associations outside the task, etc. Perception research has shown that stimulus complexity or semantic content is a key factor influencing performance across various tasks, including shape recognition, search time, discrimination, perceptual learning, and memory (Donderi, 2005; Donderi, 2006; Güçlütürk et al., 2018; Kayaert and Wagemans, 2009; Mavrides and Brown, 1969; Anderson and Leonard, 1958; Ahissar and Hochstein, 1997; Bakhtiari et al., 2020; Maor et al., 2019; Chai et al., 2010). Studies have demonstrated that higher visual complexity and saliency can enhance cortical activation and facilitate faster, more accurate recognition of faces and associations by increasing the distinctiveness of stimuli (Güçlütürk et al., 2018; Bender et al., 2017; Arslan et al., 2020). Additionally, the semantic content of the visual stimuli can affect learning performance, as shown in a recent publication by Tót and colleagues (Tót et al., 2025). To investigate the role of the semantic content of visual stimuli, a modified version of the original RAET has been developed and named Polygon test (Eördegh et al., 2022). The basic structure of the Polygon test is the same as that of the RAET, but the visual stimuli are geometric shapes, which are simpler than the faces and the fish in the original RAET. Reduced performance was found in the Polygon test in the acquisition phase, but there was no effect of stimulus semantic content on the test phase of the task (Eördegh et al., 2022).

The basal ganglia and the hippocampi are fundamentally involved in visual associative learning, and it is assumed that they also process multisensory information (Nagy et al., 2005; Nagy et al., 2006; Ravassard et al., 2013; Bates and Wolbers, 2014). Multisensory processes affect several significant areas of the brain, but in addition to the structures mentioned above, other important areas in multisensory learning include, i.e., the perirhinal cortex (Murray and Bussey, 1999), the prefrontal cortex and superior temporal sulcus (Murray et al., 2016). These structures are anatomically and functionally connected to both the basal ganglia and the hippocampus (Agster et al., 2016; Maurice et al., 1999; Deepa et al., 2022). Although the RAET only applies visual stimuli, multisensory (audiovisual) equivalence learning tests were developed and validated using the same structure as the original RAET (Eördegh et al., 2019; Tót et al., 2022), where the antecedents are well distinguishable sounds and the consequents are either the faces from the RAET (SoundFace test), or the geometric shapes from the Polygon test (SoudPolygon test). Previous results suggested that the audiovisual information only slightly influences the effectiveness in equivalence learning and the connected memory processes in healthy adults (Eördegh et al., 2019).

A fundamental question of the present study is whether the semantic content and or the modality (unimodal visual or multisensory audiovisual) of the stimuli are the primary determinants of effectiveness in associative learning. Previous results were derived from pairwise comparisons and different populations of participants. However, analyzing the learning performance in the four different tests (FaceFish, Polygon, SoundFace, SoundPolygon) in the same population of healthy individuals allows for drawing direct conclusions about the role of stimulus semantic content and stimulus modality in associative learning. The performance of the same individuals in four associative learning tests (two visual and two audiovisual, with two using visual stimuli with lower semantic content and two using visual stimuli with higher semantic content) could help assess whether the less influential factor can enhance the effect of the more influential one.

## 2 Methods

### 2.1 Participants and procedures

Altogether 117 healthy adults took part in the present study, between the age of 18–65 years (29 ± 13.89; 63 males). Prior to participation, all individuals were informed about the procedures and aims of the study and provided written informed consent. Taking part in the study was voluntary, free of any compensation and volunteers were able to quit anytime without any negative consequences. Health screening was conducted through self-report. Exclusion criteria involved any psychiatric or neurological conditions, or drugs affecting the nervous system or possibly the learning performance. Color vision was assessed using Ishihara plates to exclude participants with color vision deficiencies. Both the visual and the auditory stimuli were shown to the participants to ensure they can see or hear and differentiate them correctly.

The participants were tested individually in a silent room. They completed all four tests one after another in a pseudorandom order to avoid carry-over effect. No forced quick responses were expected to avoid time pressure.

The tests were run on laptops (Lenovo ThinkBook 15 –IIL, Lenovo, China), and headphones (Sennheiser HD439, Sennheiser, Germany) were used for the audiovisual tests.

The study protocol conformed to the Declaration of Helsinki in all respects and was approved by the Regional Research Ethics Committee for Medical Research at the University of Szeged, Hungary (27/2020-SZTE).

### 2.2 The applied stimuli in the learning tests

The participants completed computer-based visual and audiovisual learning tests developed by modifying the original Rutgers Acquired Equivalence Test introduced by Myers and colleagues (Myers et al., 2003). All tests shared the same paradigm structure, and only differed in the applied stimulus sets. The task in both the visual and the audiovisual tests is to link visual stimuli to other visual or auditory stimuli through trial-and-error learning.

**Figure 1:**
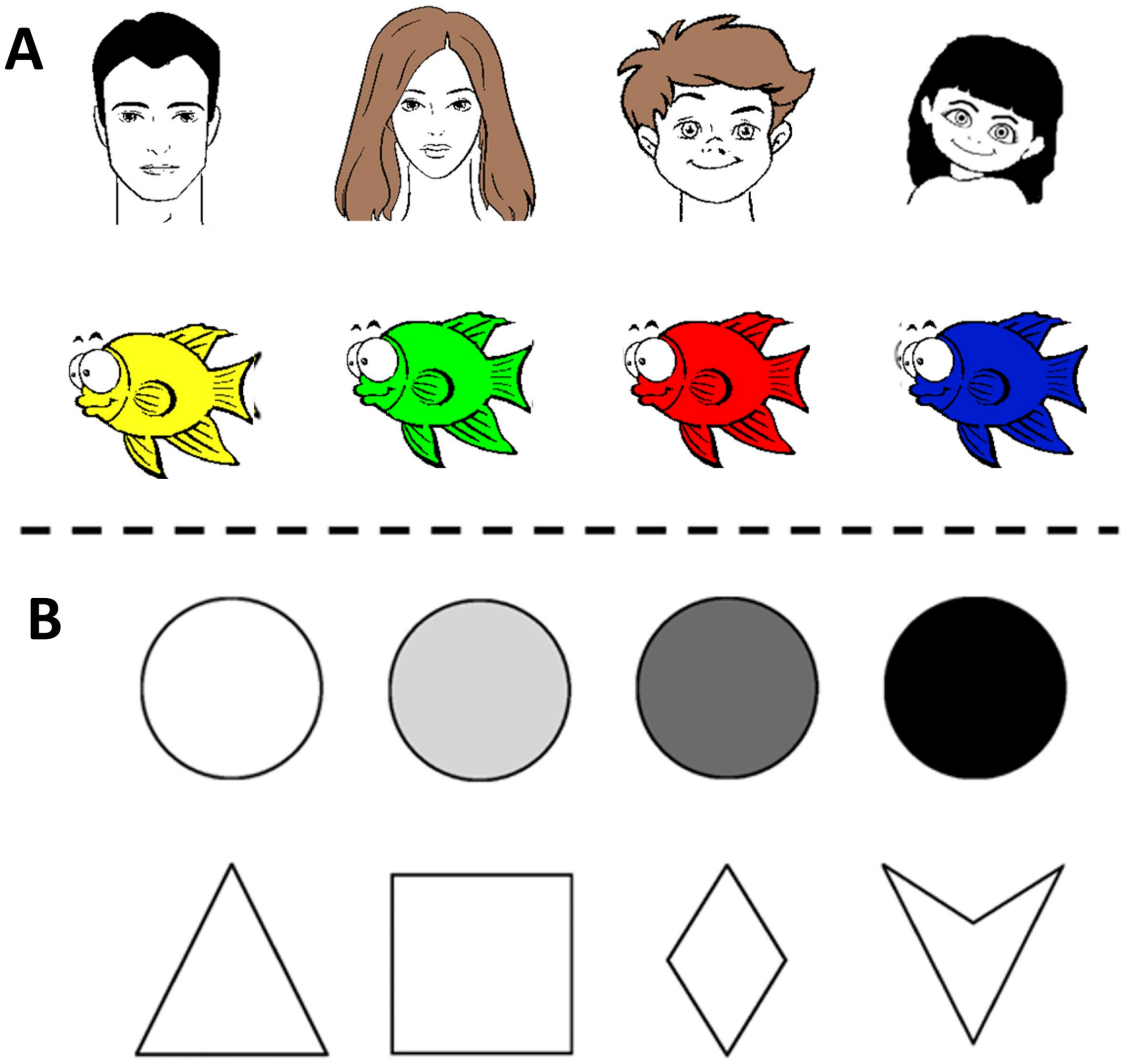
The visual stimulus sets in the applied visual tests. **(A)** In the FaceFish test, the antecedents were cartoon faces, and the consequents were different colored cartoon fish. **(B)** In the Polygon test, the antecedents were grayscaled circles, and the consequents were geometric shapes.

Two visual tests were applied, one with complex stimuli with higher semantic content (FaceFish), and one with simpler stimuli with lower semantic content (Polygon). In the FaceFish test, the antecedents were cartoon faces (a man, a woman, a boy, and a girl), and the consequents were cartoon fish in identical shape and size, but different colors (yellow, green, red, and blue). The participants had to learn which colored fish belongs to the given face (Figure 1).

In the Polygon test, the antecedents were different gray-scaled circles (white, black, light- and dark gray), while the consequents were blank two-dimensional geometric shapes (a triangle, a square, a rhombus, and a concave deltoid). The participants had to learn which geometric shape belongs to the given circle (Figure 1).

In the audiovisual tests, the antecedents were distinct sounds (a cat’s meow, a sound of a vehicle, a woman’s voice, and a guitar chord) instead of pictures. Although the auditory stimuli contained semantic information, the same four sounds were used in both audiovisual tests, thereby holding their semantic properties constant across tests. This design allowed differences between the tests to be primarily attributed to the visual stimuli. The duration of the auditory stimuli was constantly 1,500 ms. The consequents in the SoundFace test were the four cartoon faces from the FaceFish test, and in the SoundPolygon test, the same geometric shapes that were used in the Polygon test (Figure 1). Thus, one audiovisual test used more complex visual stimuli with higher semantic content, and the other used simplified visual stimuli with lower semantic content. Participants were instructed to learn which face (in SoundFace) or shape (in SoundPolygon) was associated with each sound.

Across all tasks, four antecedents (visual or auditory) and four consequents (always visual) were applied, which enabled eight possible pairings. Pairings were pseudorandomly assigned for each participant by the software.

### 2.3 The structure of the learning tests

**Figure 2:**
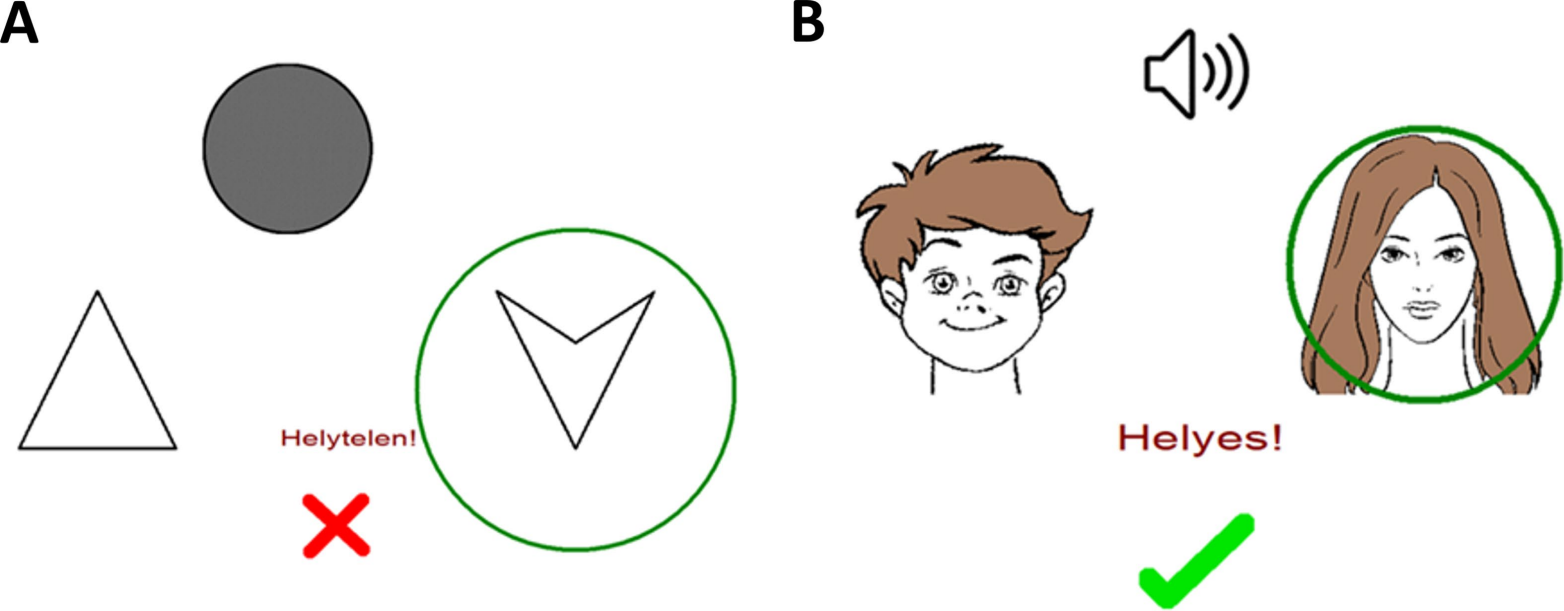
A trial in the acquisition phase in a visual **(A)** and in an audiovisual test **(B)**.

All four tasks followed the same general structure, consisting of two main phases: an acquisition phase with feedback, and a test phase without feedback. In the acquisition phase, the participant learns the stimulus pairs based on the feedback given by the software. In each trial, an antecedent (a cartoon face in FaceFish, and a gray-scaled circle in Polygon) appears at the top center of the screen, and two different consequents (fish in FaceFish, and geometric shapes in Polygon) are displayed below it on the left and right sides. In the audiovisual tests, the participants hear the antecedent sound and see the two consequents on the left and right sides of the screen simultaneously. Visual stimuli remain visible until the participants make a decision, and auditory stimuli are always played for 1,500 ms. The participants can make their choice by pressing the left or right button on a joystick. After their choice, immediate visual feedback is provided: a green checkmark with the word “Helyes!” (Correct!) if the answer is correct, and a red X with the word “Helytelen!” (Incorrect!) if the answer is incorrect (Figure 2).

In the initial steps (shaping), the participants learn two antecedent-consequent pairs (A1: X1; B1: Y1). The next step is equivalence forming: two more antecedents are presented with the same consequents (A2: X1; B2: X2), therefore the antecedents are equivalent to each other, due to sharing the same outcomes. Lastly, two more consequents are introduced (A1: X2, B1: Y2). After each newly introduced stimulus pair, the participants need to give a certain number of consecutive correct answers (4 after the first stimulus pair, then 4, 6, 8, 10, and 12 after each new stimulus pair, respectively) to proceed in the test, therefore the number of trials varies based on individual learning performance. Each antecedent is associated with two consequents, and each consequent corresponds to two antecedents, resulting eight unique antecedent–consequent pairs. Of these, only six are presented during the acquisition phase.

**Figure 3:**
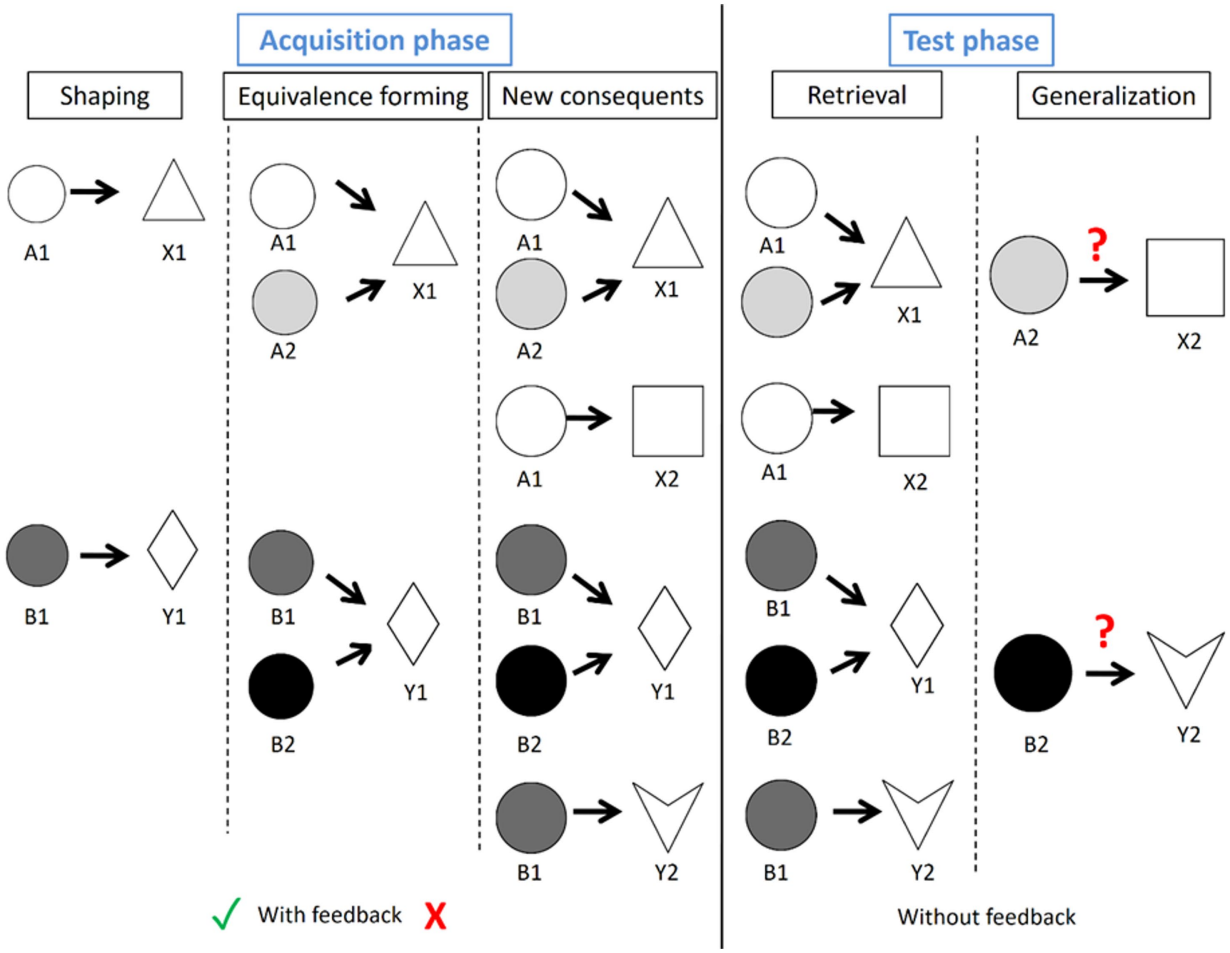
The overview structure of the Polygon test.

In the subsequent test phase, the participants have to recall the already learned stimulus pairs without any further feedback (retrieval), and the remaining two pairs (A2: X2; B2: Y2) are also presented, which are predictable if the equivalence was formed (generalization). The number of trials is constant in the test phase. There are altogether 48 trials, of which 36 are retrieval and 12 are generalization trials. The two types of trials are randomly mixed. The overall structure of the tests is shown in Figure 3.

### 2.4 Data analysis

Learning performance was characterized by four parameters. We analyzed the number of trials needed to complete the acquisition phase (NAT) and error ratios in the acquisition (AER), retrieval (RER), and generalization (GER) phases. Error ratios were calculated by dividing the number of incorrect answers by the total number of trials in the acquisition, retrieval and generalization parts.

Statistical analyses were conducted using Statistica 14.0.0.15 (TIBCO Software Inc.) and R Studio (R Core Team, 2024). As the data were non-normally distributed (Shapiro–Wilk *p* < 0.05), Friedman’s ANOVA was applied to compare the parameters in the four tests within subjects. For pairwise comparisons, Wilcoxon matched-pairs test was applied according to stimulus modality and semantic content. Effect sizes were calculated using the rank-biserial correlation (*r*), determined as r=Z/√N .

To examine the factorial structure of the within-subject design, linear mixed-effects model was fitted separately for each dependent variable. Linear mixed-effects model was specified in the general form *y* = *Xβ* + *Zb* + *ε*, where *Xβ* represented the fixed effects of modality, visual stimulus condition, and their interaction, and Zb represented participant-specific random intercepts. Participant identity was included as a random intercept to account for the dependency among repeated observations from the same individuals. Fixed effects were tested using F-tests with Satterthwaite’s approximation for denominator degrees of freedom. This approach allowed us to test the main effects and interaction while accounting for the dependency among repeated observations from the same participants.

## 3 Results

**Figure 4:**
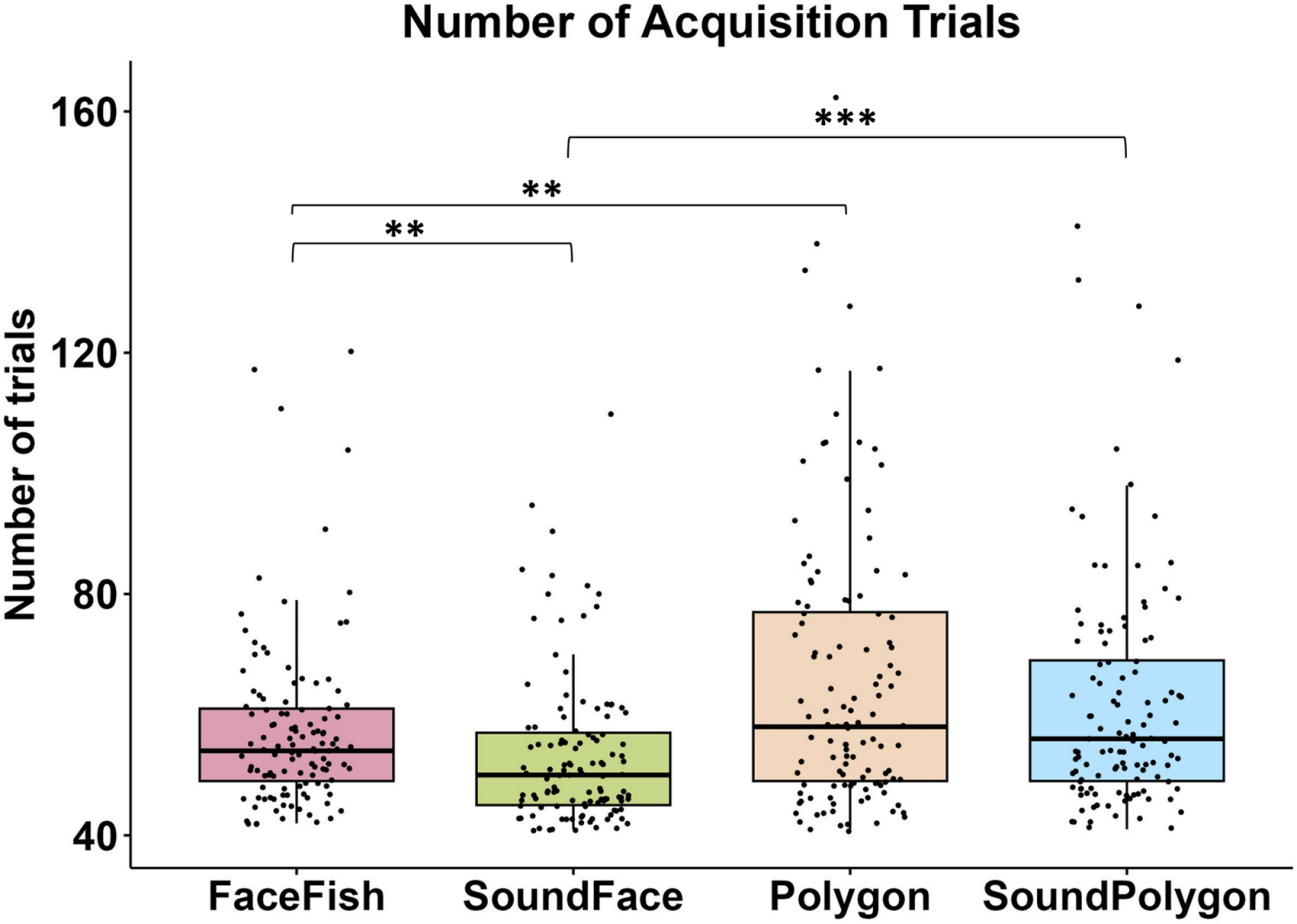
The number of trials in the acquisition phase (NAT) across the four tests. The central line within each box represents the median, the lower and upper margines of the box indicate the first (Q1) and third quartiles (Q3), respectively. The interquartile range (IQR) is the distance between Q1 and Q3. Whiskers extend to the most extreme data points within 1.5 × IQR from the quartiles. Dots represent individual data points. Asterisks indicate the significant difference: ** at the level of *p* < 0.01, and *** at the level of *p* < 0.001.

**Figure 5:**
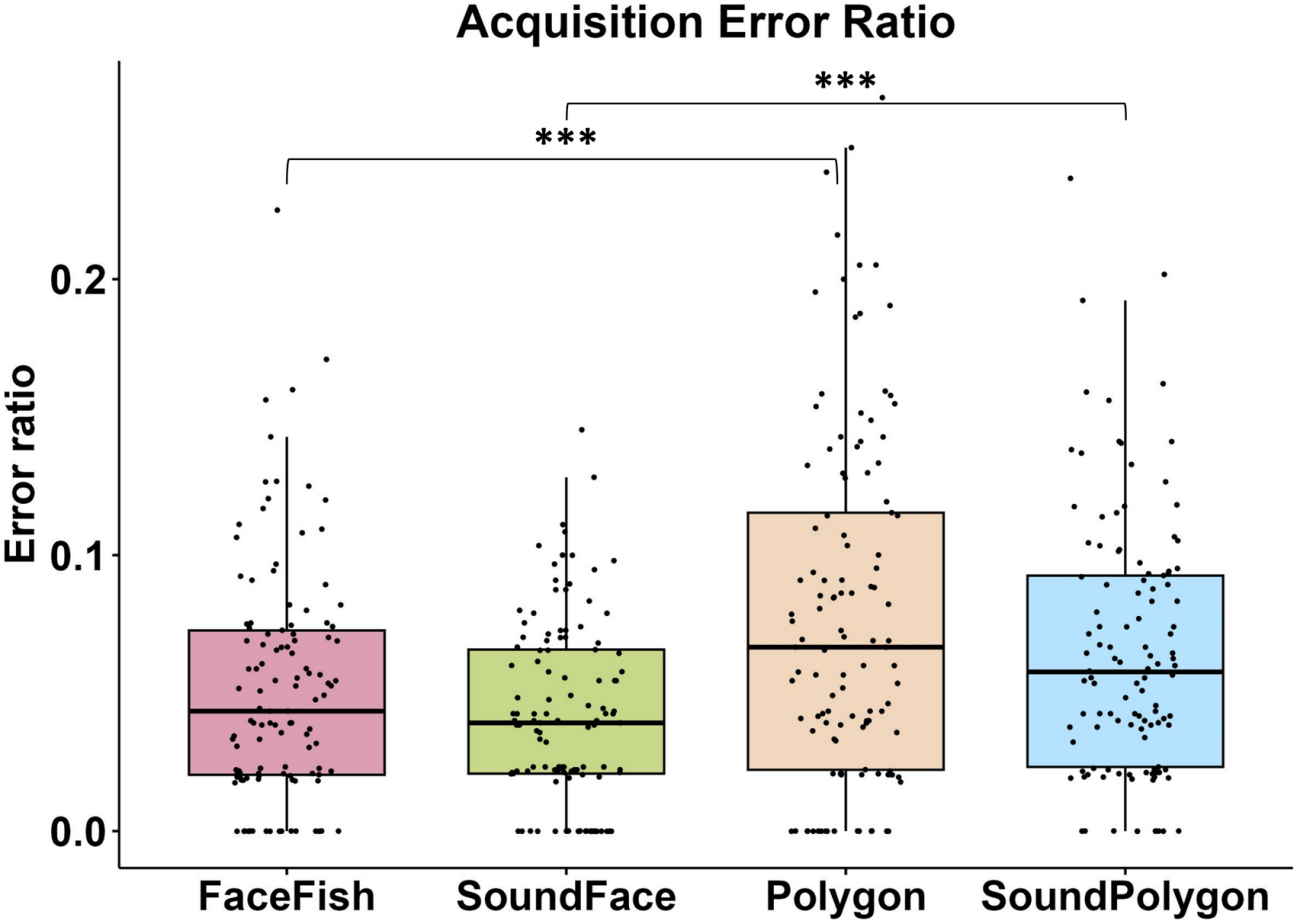
Acquisition learning error ratios (AER) across the four tests. The conventions are the same as in Figure 4.

**Figure 6:**
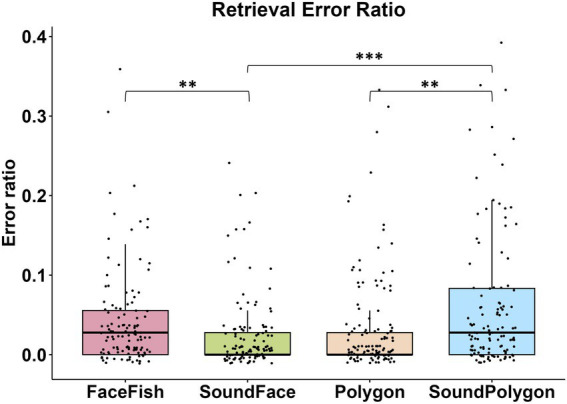
Retrieval error ratios (RER) across the four tests. The conventions are the same as in Figure 4.

**Figure 7:**
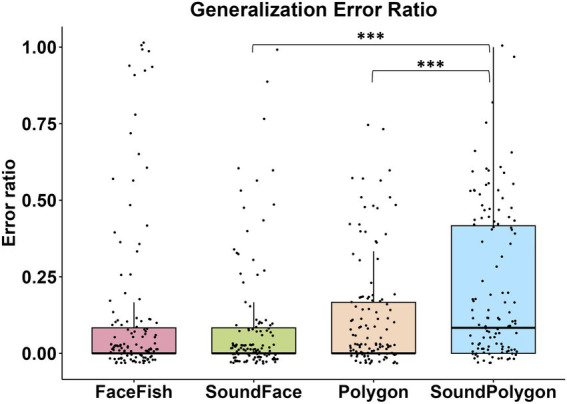
Generalization error ratios (GER) across the four tests. The conventions are the same as in Figure 4.

In the current study, we present the performance of 117 healthy adult participants in four (two visual and two audiovisual) associative equivalence learning tasks. The Friedman’s ANOVA test showed a significant difference (*p* < 0.05) among the tests in all of the analyzed parameters (number of acquisition trials, NAT; acquisition error ratio, AER; retrieval error ratio, RER; and generalization error ratio, GER). Descriptive results are shown in Table 1 and Figures 4–7. The results of the pairwise comparisons are presented in the following sections according to modality and stimulus semantic content, and the differences are also marked on Figures 4–7.

**Table 1:** Descriptive statistics of the analyzed learning parameters, *N* = 117.

Parameter	Median	Q1	Q3	Median	Q1	Q3
	FaceFish			SoundFace		
NAT	54	49	61	50	45	57
AER	0.043	0.020	0.073	0.039	0.021	0.066
RER	0.028	0.000	0.056	0.000	0.000	0.028
GER	0.000	0.000	0.083	0.000	0.000	0.083

Parameter	Median	Q1	Q3	Median	Q1	Q3
	Polygon			SoundPolygon		
NAT	58	49	77	56	49	69
AER	0.067	0.022	0.115	0.058	0.023	0.093
RER	0.000	0.000	0.028	0.028	0.000	0.083
GER	0.000	0.000	0.167	0.083	0.000	0.417

### 3.1 The results of linear-mixed-effects model

To directly compare the effects of stimulus semantic content and modality on associative learning and the connected memory processes, linear mixed-effects model was fitted separately for each dependent variable (NAT, AER, RER, and GER). Fixed effects were tested using F-tests with Satterthwaite’s approximation for denominator degrees of freedom. The results of the statistical analysis can be seen in Table 2.

**Table 2:** Results of the linear mixed-effects model analysis.

Parameter	Effect	dF	*F*	*p*
NAT	Semantic content	1	29.916	0.000
	Modality	1	7.770	0.006
	Interaction	1	0.010	0.921
AER	Semantic content	1	36.241	0.000
	Modality	1	8.190	0.004
	Interaction	1	0.115	0.735
RER	Semantic content	1	7.567	0.006
	Modality	1	0.303	0.583
	Interaction	1	12.637	0.000
GER	Semantic content	1	7.498	0.006
	Modality	1	1.064	0.303
	Interaction	1	12.647	0.000

The mixed-effects model revealed that stimulus semantic content had a statistically significant effect on all investigated parameters: NAT (*F*(1, 351) = 29.916, *p* < 0.001), AER (*F*(1, 351) = 36.241, *p* < 0.001), RER (*F*(1, 351) = 7.567, *p* = 0.006), and GER (*F*(1, 351) = 7.498, *p* = 0.006). In contrast to these findings, stimulus modality had a significant effect only in the acquisition phase (NAT: *F*(1, 351) = 7.770, *p* = 0.006; AER: *F*(1, 351) = 8.190, *p* = 0.004), but not in the test phase (RER: *F*(1, 351) = 0.303, *p* = 0.583; GER: *F*(1, 351) = 1.064, *p* = 0.303). This consistent pattern across all task phases suggests that stimulus semantic content may have a broader and more robust influence on associative equivalence learning performance than stimulus modality.

Furthermore, analysis of interactions between stimulus semantic content and modality revealed significant interactions exclusively in the test phase (RER: *F*(1, 351) = 12.637, *p* < 0.001; GER: *F*(1, 351) = 12.647, *p* < 0.001), but not in the acquisition phase (NAT: *F*(1, 351) = 0.010, *p* = 0.928; AER: *F*(1, 351) = 0.115, *p* = 0.759). These findings indicate that modality’s effect in the test phase may have been conditional on visual stimulus semantic content. Specifically, audiovisual presentation was associated with improved retrieval and generalization (*p* < 0.05) only when paired with more complex visual stimuli with higher semantic content (SoundFace vs. FaceFish), while performance was significantly worse (*p* < 0.05) when modality was combined with simplified visual stimuli with lower semantic content (SoundPolygon vs. Polygon). This interaction does not imply that modality enhances semantic content generally, but rather that audiovisual input provided a benefit only under specific, circumscribed conditions. These effects have been examined further in the pairwise comparisons below, where the detailed statistical analysis is also presented.

### 3.2 The effect of modality on learning and the connected memory processes

Between those visual and audiovisual tests that applied complex visual stimuli with higher semantic content (FaceFish vs. SoundFace), the NAT was lower in the SoundFace test (*Z* = 2.788, *p* = 0.005, *r* = 0.261), but the AERs were not significantly different (*Z* = 1.782, *p* = 0.075, *r* = 0.168).

In the test phase, the RER was significantly lower in the SoundFace test than in the FaceFish test (*Z* = 2.772, *p* = 0.006, *r* = 0.322), but there was no difference in the GER (*Z* = 0.100, *p* = 0.318, *r* = 0.120) between the two tests.

Comparing those visual and audiovisual tests in which simplified visual stimuli were applied (Polygon vs. SoundPolygon), there was no significant difference in the acquisition phase between the two tests, neither in NAT (*Z* = 1.669, *p* = 0.095, *r* = 0.156) nor in AER (*Z* = 1.298, *p* = 0.194, *r* = 0,122). On the contrary, the performance in the test phase was significantly poorer in the audiovisual SoundPolygon test than in the visual Polygon test, both in RER (*Z* = 2.602, *p* = 0.009, *r* = 0.291) and GER (*Z* = 3.573, *p* < 0.001, *r* = 0.392).

### 3.3 The effect of semantic content of visual stimuli on learning and the connected memory processes

Comparing the two visual tests (FaceFish vs. Polygon) with different stimulus semantic content, the participants learned significantly worse in the Polygon test. The difference was significant in the acquisition phase, both in NAT (*Z* = 3.087, *p* = 0.002, *r* = 0.290) and AER (*Z* = 3.725, *p* < 0.001, *r* = 0.352). However, there was no significant difference in RER (*Z* = 0.765, *p* = 0.444, *r* = 0.084) or GER (*Z* = 0.347, *p* = 0.728, *r* = 0.040) in the test phase.

In case of the two audiovisual tests (SoundFace vs. SoundPolygon), the participants performed better in the SoundFace test, both in the acquisition phase (NAT: *Z* = 4.233, *p* < 0.001, *r* = 0.398; AER: *Z* = 4.012, *p* < 0.001, *r* = 0.376) and in the test phase (RER: *Z* = 4.045, *p* < 0.001, *r* = 0.444; GER: *Z* = 4.345, *p* < 0.001, *r* = 0.471).

## 4 Discussion

To our knowledge, this is the first systematic study to investigate the effects of stimulus modality (visual vs. audiovisual) and the effects of semantic content of visual stimuli on associative equivalence learning simultaneously in the same relatively large healthy adult human population. Our results indicate that both stimulus modality and the characteristics of the visual stimuli influenced performance in associative equivalence learning, but semantic content may have a stronger influence on the learning performance in healthy humans.

This was supported by both non-parametric comparisons and linear mixed-effects model, which accounted for the repeated-measures structure of the data and revealed significant effects associated with semantic content on all performance metrics, whereas modality showed a significant influence mainly in the acquisition phase. Although we observed significant interactions between semantic content and modality in both parts of the test phase (retrieval and generalization), these interactions should not be interpreted as evidence that audiovisual presentation generally enhances learning. Rather, the effect of modality appeared to depend on the characteristics of the visual stimuli with which auditory information was paired. Audiovisual presentation was associated with favorable performance when combined with more complex and distinctive visual stimuli with higher semantic content. In contrast, when auditory stimuli were paired with visual stimuli with lower semantic content, modality did not provide a comparable advantage and was associated with weaker test-phase performance in some measures. This pattern suggests that the contribution of modality is context-dependent and may rely on the availability of sufficiently distinctive and informative visual stimulus representations.

Besides the original visual equivalence learning test, the RAET, in which different schematic faces are used as antecedents and different colored fish as consequents (Myers et al., 2003; Öze et al., 2017), a test was developed applying the same structure, but using simplified visual stimuli (grayscaled circles as antecedents and polygons as consequents; the Polygon test), where the stimuli are visually simpler and have lower semantic content, to gain a more detailed picture of the cognitive functions of healthy humans. Previous results in healthy adults showed that the lack of extra cues (such as color, small details of the figures) affect performance in the acquisition phase, but not in the test phase (Eördegh et al., 2022). One possible interpretation is that stimuli with higher semantic content and more distinctive visual features may support visual or verbal encoding more effectively, leading to richer memory representations (Paivio, 1990; Bi, 2021; Kounios, 2002), which may be reflected in better learning performance in the present study. We argue that higher visual semantic content may provide more opportunities to acquire the associations. The more distinctive features of visual stimuli may facilitate the recognition of the equivalence rule, thereby improving the formation of associations (Ullman et al., 2016; Craik and Lockhart, 1972). Despite these advantages in the equivalence learning phase, the semantic content of the visual stimuli did not necessarily influence test phase performance (both retrieval and generalization functions). This suggests that while visual stimuli with higher semantic content may enhance the initial encoding of information, they may also introduce greater cognitive memory load during retrieval, potentially making it more difficult to access the stored information (Sweller, 2011; Baddeley, 2000).

Since the basal ganglia and the hippocampi are not only involved in visual equivalence learning and the connected memory processes, but in multisensory information processing as well (Nagy et al., 2005; Nagy et al., 2006; Ravassard et al., 2013; Bates and Wolbers, 2014; Chudler et al., 1995; Lee et al., 2017; Schwarz et al., 1984), audiovisual versions (SoundFace and SoundPolygon) of the RAET and Polygon tests had also been developed. During multisensory integration, stimuli of different modalities together produce a neural response that is significantly different from that elicited by each stimulus alone (Stein and Meredith, 1993). This modality interaction plays essential role in cognitive processes (Talsma, 2015), for example visual perception (Frassinetti et al., 2002), object recognition (Fort et al., 2002; Suied et al., 2009), recognition of emotional changes (Chen et al., 2016), face and voice recognition (Love et al., 2011), or in person recognizing (Joassin et al., 2011). It affects the reaction time and accuracy of answers and the perceived threshold as well (Hershenson, 1962; Miller, 1982; Patching and Quinlan, 2004). However, because visual consequents were maintained in all four task versions, visual information was present across all conditions. Therefore, the interpretation of modality effects is more limited than a fully visual-versus-auditory design would allow. In the present study, modality effects should be interpreted as reflecting the addition of auditory antecedents while retaining visual consequents, rather than as a comparison between purely visual and purely auditory associative learning.

A previous study with 151 healthy adult volunteers (Eördegh et al., 2019) demonstrated that visual (RAET) and multisensory (SoundFace) associative learning are not significantly different, although performance is slightly better in the audiovisual SoundFace test. Analogous to this study, it was recently demonstrated that performance in the acquisition phase is not different between the visual and the audiovisual tests using simpler visual stimuli (Polygon vs. SoundPolygon). Interestingly, the audiovisual stimuli could elicit weaker performance in both the retrieval and generalization parts of the test phase (Tót et al., 2025). This suggests that in the absence of complex visual stimuli with higher semantic content, the benefits of modality become less evident and may even hinder performance. It seems that the addition of auditory information in the retrieval and generalization parts of the test phase may introduce unnecessary interference when processing simple visual stimuli as it was found in a recent study (Tót et al., 2025). The last pairwise comparison was performed between the two audiovisual tests (SoundFace vs. SoundPolygon), where all of the learning parameters (both in the acquisition and the test phases) were significantly better in the test which applied more complex visual consequent stimuli with higher semantic content (Tót et al., 2022).

The present study represents, to our knowledge, the first systematic investigation of stimulus modality and semantic content within the same relatively large participant sample. A strength of the present study is that it reproduces and extends previous findings derived from pairwise comparisons in smaller samples (Eördegh et al., 2022; Tót et al., 2022; Tót et al., 2025). While previous studies reported pairwise comparisons between specific task versions, the present within-subject design allowed all four tasks to be compared directly. Thus, the novel contribution of this study is the integration of previous findings into a single experimental framework, enabling a more direct evaluation of the relative contribution of modality and visual stimulus characteristics to associative equivalence learning.

The results indicated that both modality and semantic content may have a significant effect on performance in equivalence learning, but the effects associated with the visual stimulus sets were more robust than those associated with modality alone. This interpretation is also supported by a previous study on semantic association (Tót et al., 2025), in which the semantic content of the visual stimuli used in these tasks was directly examined. In that study, participants generated associations to face, fish, and polygon stimuli, and the number of unique associations was used as an index of semantic content. Face and fish stimuli showed higher semantic content than polygon stimuli; however, audiovisual associative learning performance was consistently better with face stimuli than with fish or polygon stimuli. This indicates that semantic content alone is not sufficient to explain the observed learning differences. Instead, other visual stimulus properties, such as the number and variability of distinctive features, visual complexity, salience, and discriminability, may also contribute substantially to performance. This does not exclude the multisensory integration theory, which suggests that combining auditory and visual cues can enhance learning efficiency by providing additional contextual information and can facilitate stimulus encoding (Shams and Seitz, 2008; Eördegh et al., 2022; Xie et al., 2017; Heikkilä et al., 2015). Similarly, the less influential factor (in this case, stimulus modality) may significantly enhance the effect of the more influential factor (in this case, semantic content of visual stimuli), particularly in the retrieval and generalization functions. Our overall findings suggest that the characteristics of the visual stimuli may have a stronger influence on associative equivalence learning than stimulus modality alone. This may also be relevant for clinical applications of acquired equivalence tasks. Because different task phases and stimulus formats may place different demands on associative learning, retrieval, generalization, multisensory processing, and memory-related mechanisms, performance may vary depending on both the stimulus set and the clinical population examined. Thus, comparing task variants that differ in modality and visual stimulus characteristics may help identify which types of information different populations can use most effectively during associative learning.

Finally, we have to mention some shortcomings of the study and the present findings should be interpreted in the context of the stimulus characteristics used in the tasks. Importantly, because semantic content was not manipulated independently from other stimulus properties, these findings should not be interpreted as demonstrating a sole, isolated effect of semantic content. Rather, they suggest that the overall quality and characteristics of the visual stimuli, including semantic content, visual complexity, color, perceptual salience, familiarity, discriminability, and potential verbalizability, may be particularly important determinants of performance in associative equivalence learning. Therefore, the present results should be interpreted as showing that performance differs across stimulus sets that vary simultaneously in multiple perceptual and semantic characteristics, rather than as evidence for an isolated effect of semantic content. It is important to distinguish the empirical findings of the present study from possible theoretical interpretations. Empirically, our results show that performance differed across task versions that varied in stimulus modality and in several characteristics of the visual stimuli. However, the present design does not directly test the cognitive mechanisms underlying these differences. Therefore, explanations involving enhanced encoding, richer memory representations, increased retrieval-related cognitive load, or audiovisual interference should be considered possible interpretations rather than direct conclusions. Future studies using experimental designs that independently manipulate these factors and directly assess encoding, memory load, and interference processes are needed to evaluate these mechanisms more precisely. Furthermore, the present study only investigated healthy adults; thus, the information about the development of the associative learning remains unclear in the case of modality and stimulus semantic content. In contrast to adults, audiovisual information with visual stimuli with more semantic content, could strongly help the associative learning in healthy children and adolescents (Eördegh et al., 2022). The question is whether the same phenomenon can be observed using simpler visual stimuli in children and adolescents as well. Another limitation of the present study is the relatively homogeneous composition of the participant sample. Most participants were university students or individuals with a university degree, and the age range was concentrated primarily between 18 and 25 years. This limited variability in age and education level may constrain the generalizability of our findings to the broader populations. In future studies, it would be beneficial to recruit participants with a more diverse range of educational backgrounds and a broader age distribution. This would allow for the investigation of how individual-level variables, such as age, educational attainment, or cognitive ability may moderate the effects of stimulus modality and semantic content on associative learning. Another shortcoming of the study is that it investigated only audiovisual stimuli, and did not focus on other sensory modalities (i.e., somatosensory). It is shown that somatosensory information is also represented in the basal ganglia (Nagy et al., 2005; Nagy et al., 2006). These subcortical structures are involved in equivalence learning; thus, investigating the somatosensory modality may represent a promising direction for future research aimed at describing the modality dependence of associative equivalence learning more accurately in humans. Additionally, the application of more than two (one with higher and one with lower semantic content) visual stimulus sets could help further refine the effect of visual stimulus semantic content in equivalence learning tasks.

In summary, our findings suggest that visual stimulus characteristics show a stronger and more consistent association with associative equivalence learning performance than stimulus modality alone. Audiovisual presentation did not uniformly improve learning; rather, its effect appeared to depend on the complexity, distinctiveness, and semantic content of the visual stimuli with which auditory information was paired. These results underscore the importance of visual stimulus quality in supporting associative learning and provide a more nuanced view of multisensory contributions, suggesting that multisensory input can be helpful but it is not uniformly advantageous.

## Data Availability

The raw data supporting the conclusions of this article will be made available by the authors, without undue reservation.
